# Discretization of Gene Expression Data Unmasks Molecular Subgroups Recurring in Different Human Cancer Types

**DOI:** 10.1371/journal.pone.0161514

**Published:** 2016-08-18

**Authors:** Manfred Beleut, Robert Soeldner, Mark Egorov, Rolf Guenther, Silvia Dehler, Corinna Morys-Wortmann, Holger Moch, Karsten Henco, Peter Schraml

**Affiliations:** 1 Qlaym Healthcare AG, Hans-Adolf-Krebs Weg 1, 37077 Goettingen, Germany; 2 Cancer Registry Zurich and Zug, University Hospital Zurich, Zurich, Switzerland; 3 Institute of Surgical Pathology, University Hospital Zurich, Schmelzbergstrasse 12, 8091 Zurich, Switzerland; Fondazione IRCCS Istituto Nazionale dei Tumori, ITALY

## Abstract

Despite the individually different molecular alterations in tumors, the malignancy associated biological traits are strikingly similar. Results of a previous study using renal cell carcinoma (RCC) as a model pointed towards cancer-related features, which could be visualized as three groups by microarray based gene expression analysis. In this study, we used a mathematic model to verify the presence of these groups in RCC as well as in other cancer types. We developed an algorithm for gene-expression deviation profiling for analyzing gene expression data of a total of 8397 patients with 13 different cancer types and normal tissues. We revealed three common Cancer Transcriptomic Profiles (CTPs) which recurred in all investigated tumors. Additionally, CTPs remained robust regardless of the functions or numbers of genes analyzed. CTPs may represent common genetic fingerprints, which potentially reflect the closely related biological traits of human cancers.

## Introduction

The use of DNA microarray technologies enabled the generation of myriads of data, sustaining further molecular sub-classification of many previously described pathologic phenotypes with significant effects on clinical decision making and prognosis [1–6]. In particular, gene expression analysis served as an efficient cancer sub-classification tool [7], and is regarded as the most downstream signal onto which accumulated effects from different molecular layers such as genomics, proteomics or methylomics may imprint [8]. Depictions of distinct driver mutations in genes such as *BRAF*, *EGFR*, *PAK5*, *HER2*, *ALK* or hormone receptors [9, 10], all of which are embedded in individual tumor specific mutational landscapes [11–13], have also been used as prognostic or therapeutic biomarkers for further patient stratification of different cancer subtypes [14–17].

The fact that close to 75% of all genes have already been identified as being potentially cancer relevant [18], and the unique molecular make-up of each tumor [19] suggest that cancer evolution and progression are complex processes. In order to better understand the biology of tumors, functionally classifying deregulated gene candidates according to specific biologic processes is often the practice of choice [20]. Despite the unique molecular background of each malignant tumor, the “Hallmarks of Cancer” [21] claim that all malignant neoplasms acquire similar characteristics, enabling the transformation from a normal to a cancer cell.

In line with the proposed concept of cancer hallmarks, we assumed that regardless of their highly diverse phenotypic and genotypic appearance, all tumors share common cancer traits, which may potentially be detected *via* gene expression analysis. This hypothesis is backed by our recent identification of global gene expression outputs of prognostic relevance in renal cell carcinoma (RCC) [22]. Based on this finding we evaluated whether similar gene expression patterns may exist also in other cancer types.

Unbiased by any cancer-specific marker or classification schemes currently used, we analyzed 55 published studies of gene expression data encompassing a total of 8397 patients with 13 different cancer types by means of gene-expression deviation profiling.

## Materials and Methods

### RCC patient data

**Fig 1:**
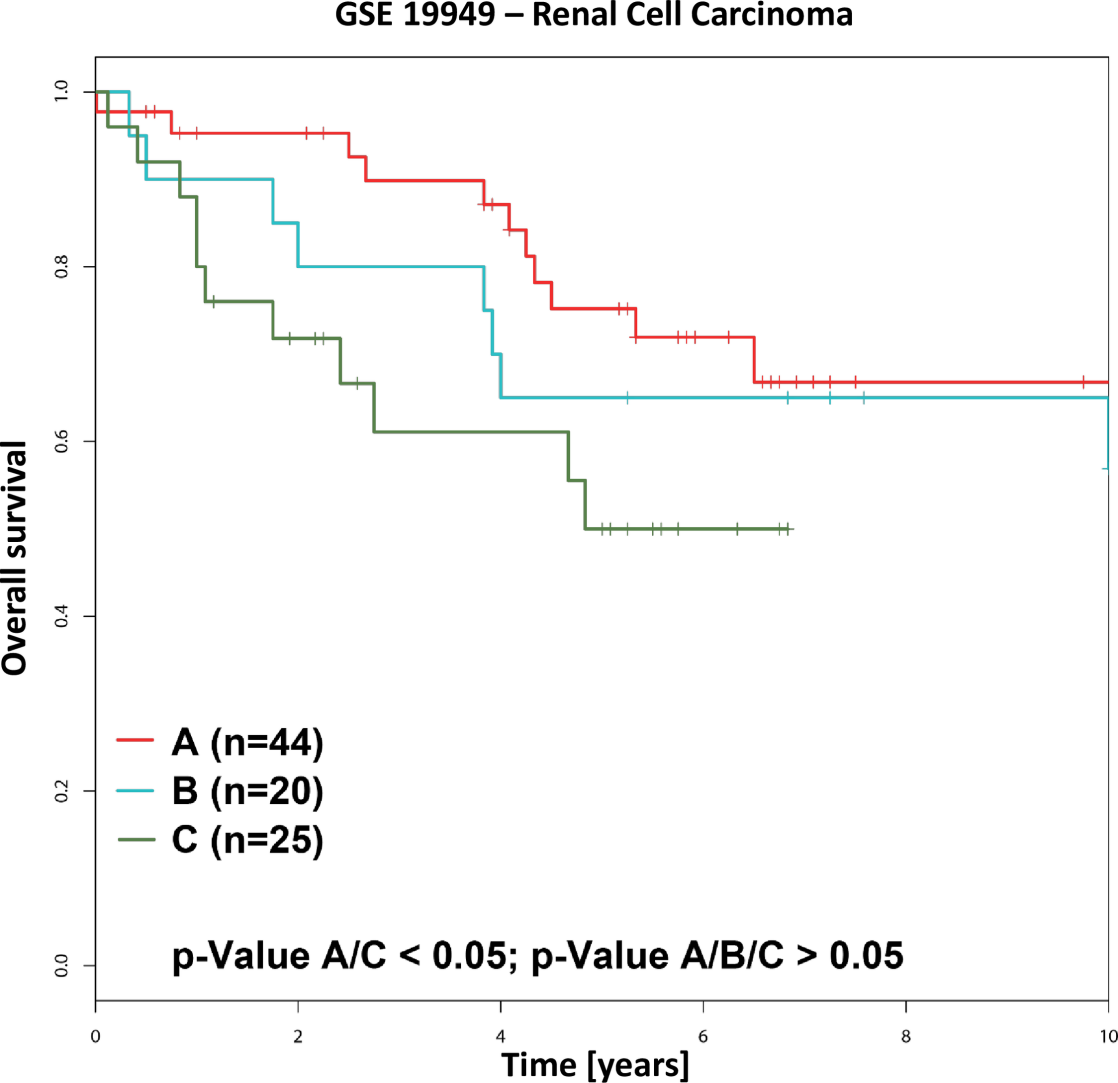
**RCC groups A, B, and C and their correlation with patient survival.** Kaplan-Meier curves for overall survival in relation to cancer transcriptome profiles (CTPs) as described for the patient cohort GSE19949 [22]. Survival data were made available by the Cancer Registry Zurich and Zug.

Survival data linked to the published dataset GSE19949 was provided by the Cancer Registry Zurich and Zug and approved by the ethics committee of the Canton Zurich (KEK-ZH-Nr. 2013–0629). This data was used for the verification of the prognostic relevance (Fig 1) as proposed from our previous work using the same patient cohort [22].

### Gene expression data and normalization

All data was retrieved from the GEO repository as published by the authors. An overview of all analyzed datasets is given in S1 Table. Data were pre-processed and normalized as described in S1 Text. Table A in S1 Text and S2 Table provide a systematic overview of all analyzed datasets including clinic-pathological data.

### Model generation and algorithm development

The method comprised the following steps: For every normalized log_2_ gene probe set value, the mean expression value was calculated over all samples of the given data set. This mean value was then subtracted from the log_2_ expression values of all samples. The calculated difference denoted the individual deviance of the sample from the mean expression value for the respective probe set.

To those expression values which were close to mean the value 0, to those with significantly higher values than mean the value 1, and to those with significantly lower expression than mean the value -1 were assigned. The threshold for high and low was set to 43% of the standard deviation, which means that all three values (-1, 0, 1) occurred at almost the same frequency. The value 0 was assigned to those deviation values which were located between -0,43 σ and +0,43 σ. Deviation values lower than -0,43 σ were assigned with -1, those higher than +0,43 σ with +1. Additional categories were therefore automatically excluded.

The samples were then clustered using well known clustering methods such as k-means or SOM, so that samples with similar (individual) profiles were assigned to the same group A, B, or C. In order to generate the respective CTPs the average values for each probe set and group were calculated.

**Fig 2:**
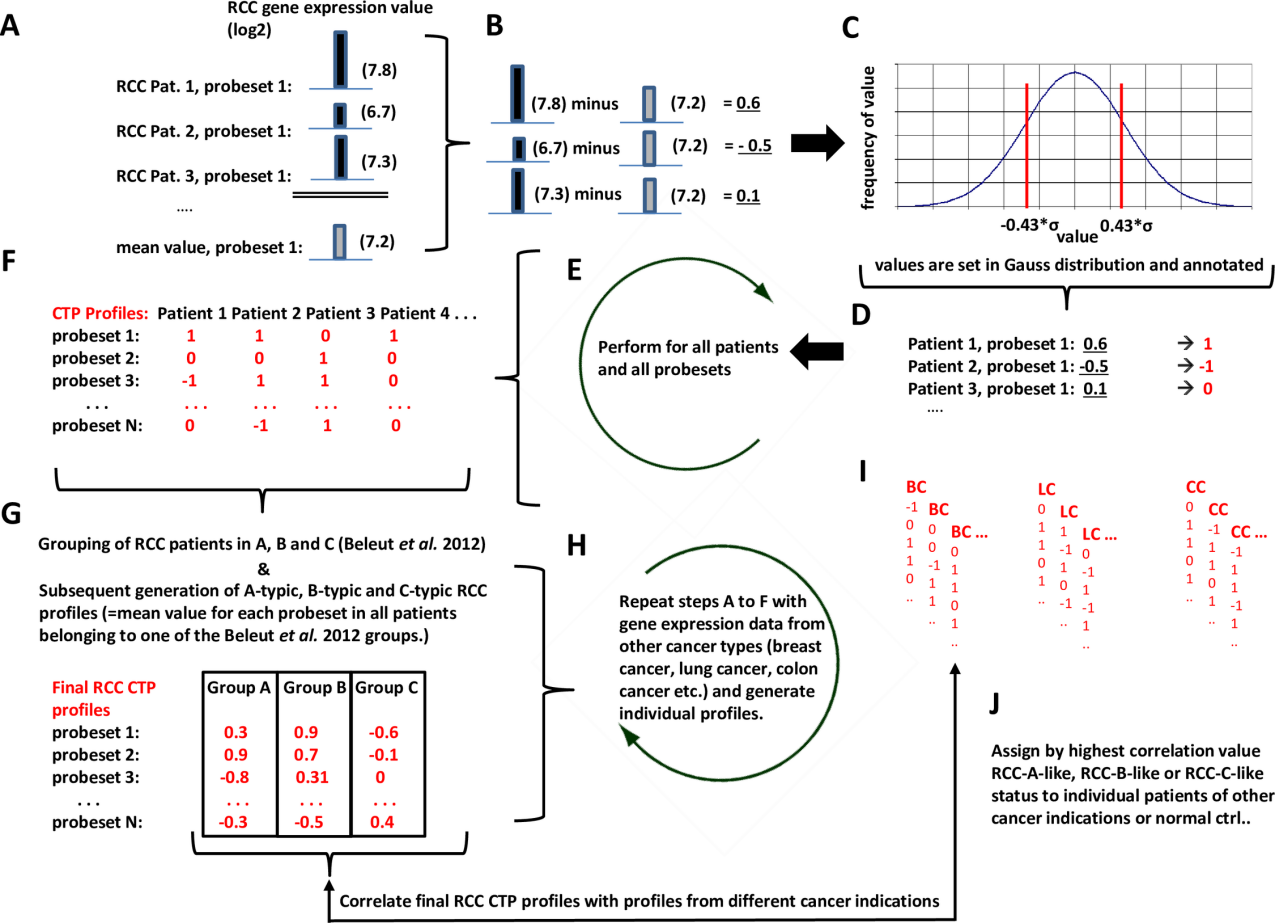
Workflow for generating RCC-CTPs. **A**. Determination of the gene expression value (log_2_) of gene probe set 1 in all RCC samples tested and generation of the mean value for probe set 1 of the entire RCC tumor cohort (exemplified values are shown). **B.** Subtraction of the mean value from true expression value for probe set 1 of each patient. **C.** Distribution of the remaining deviation values from mean for probe set 1. **D.** Annotation of remaining deviation values from mean for probe set 1 as 1, -1 or 0 depending on their localization in the distribution. **E.** Steps A to D are performed for all probe sets of the gene expression microarray. **F.** Individual CTP profile for each patient given by a vector covering all expression values. **G.** Grouping of RCC patients into CTP-A, -B or -C according to Beleut *et al*. 2012; Determination of the CTP mean values of all gene probe sets for patient group A, group B and group C and generation of the final RCC CTP A, -B and -C target vectors. **H.** Gene expression data from other cancer types calculated according to steps A to F and generation of patient-specific CTP-vectors. **I.** Correlation of RCC CTP-A, -B and -C target vectors from step G with patient-specific CTP of other cancer types derived from step H. (BC) breast cancer patients; (LC) lung cancer patients, (CC) colon cancer patients. **J.** Assigning a patient or control to tumor subgroup according to the CTP with the highest correlation.

At the end of this procedure, a whole genome CTP profile existed for every group, represented by a vector with a length equal to the number of probe sets of the respective microarray and values ranging between -1 and 1 (Fig 2 and S1 Text).

For the RCC data set GSE19949 the assignments to A, B, or C groups were known *a priori* [22]. Detailed methodological descriptions and codes can further be found in S1 Text. For the interested reader, raw and calculated data is available upon request.

### *De Finetti*-like mappings

As proposed by *de Finetti* [23] ternary plots or *de Finetti* mappings are efficient means to depict percentage compositions for 3 parameters in an equilateral triangle [24, 25]. Using this method, we were able to plot the distances of individual patient samples obtained from the 3 predefined CTP centroids. Additional info to the *de Finetti* like mappings can be found in S1 Text.

### CTP assignment

**Fig 3:**
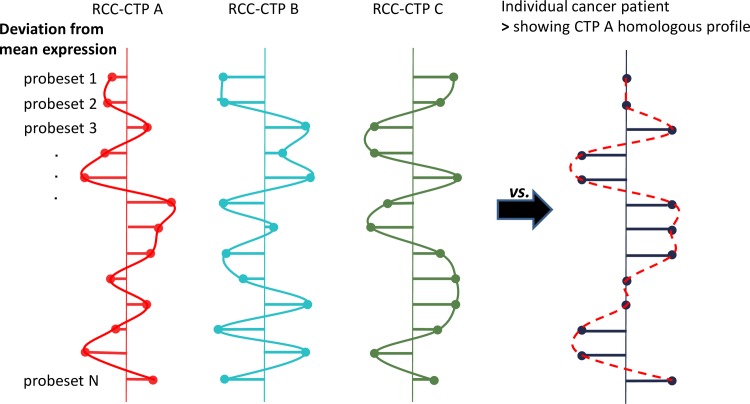
Graphical illustration of CTPs and patient classification. Shown is a graphical overview on the nature of generated RCC-CTPs as well as their comparison with the CTP of one patient with a different cancer type. The deviation from the mean expression for all probe sets and their relative correlation to each other (continuous curves) define the RCC-CTP target profiles. For CTP assignment, the CTP profile of an individual patient with another cancer type (dashed curve) is compared to the target RCC-CTPs.

For each sample of a new data set, the expression profile was calculated as mentioned above. As for individual samples no averaging takes place, the profiles contained only the values -1, 0, or 1. The correlations between the sample profile and the whole genome A-, B- and C-CTPs were calculated. The sample was assigned to the CTP with the highest Pearson correlation (Figs 2 and 3 and S1 Text).

## Results

### Genome wide expression analysis confirms three subgroups in two independent RCC patient cohorts

By analyzing gene expression profiles in a RCC patient cohort (GSE19949) we recently identified three subgroups (termed groups A, B and C), which were not significantly associated with pathological prognostic parameters such as nuclear differentiation grade and tumor stage (Beleut *et al*., see additional file 12, table S8 [22]). As survival data was sparse at that time point for this patient cohort, we analyzed a second RCC patient cohort using tissue microarrays and immunohistochemistry. By correlating survival data and expression levels of proteins whose genes were highly expressed in the three groups, we found an association between identified subgroups and patient clinical outcome [22].

As survival data were meanwhile also available for 89 patients of the first cohort, we could confirm the result obtained from the second tissue microarray patient cohort. As shown in Fig 1 the survival rate was highest for patients belonging to group A followed by group B and group C, with statistically significant differences between group A versus group C (p-value = 0.040).

### Algorithmic Cancer Transcriptomic Profile (CTP) model for subgrouping RCC

To exclude that those RCC gene-expression signatures are biased due to non-cancerous effects, we first aimed at designing a comprehensive algorithmic Cancer Transcriptomic Profile (CTP) classification model using the RCC gene expression data. Requirements for the model included automated reassignment of GSE19949 RCC tumors to the known CTP groups A, B and C. Additionally, the algorithms should be independent from biases derived from different normalization techniques as well as tissue type artefacts. Finally, the model should consider the entire gene expression profile resulting from the microarray chip.

In order to further investigate whether RCCs could be sub-classified by our proposed CTP-model, we utilized the following approach. We classified the GSE19949 tumors into the three known groups by implementing the algorithmic workflow shown in Fig 2A–2G.

Fig 3 provides a graphical overview on the nature and comparison of CTPs within RCC (Fig A in S1 Text). Highest Pearson correlation with one of the three RCC-CTPs assigned a patient either to CTP group A, B or C.

**Fig 4:**
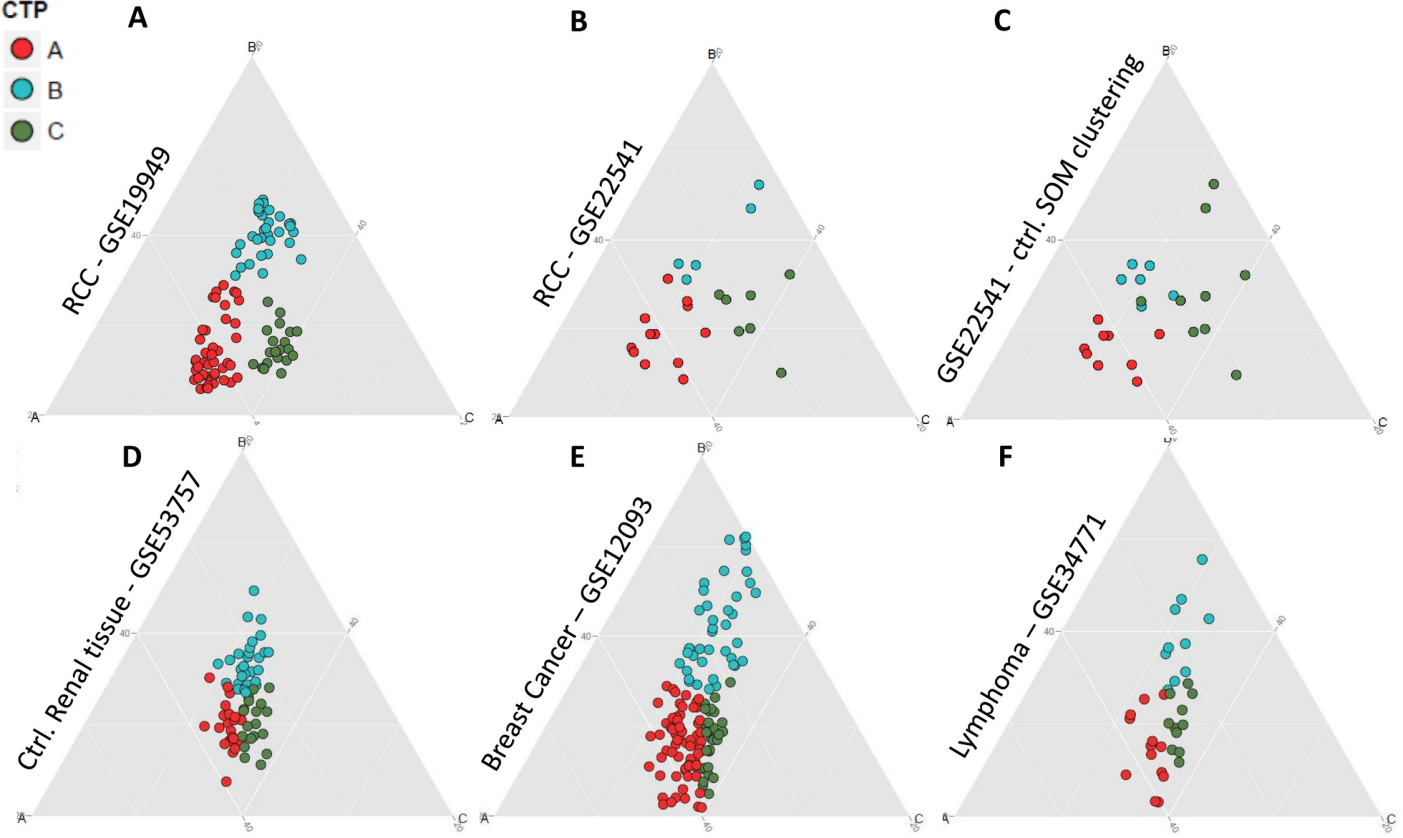
Visualization of CTPs by *de Finetti* like mapping. **A.** *De Finetti* like diagram illustrating RCC classification of GSE19949 into the three CTP groups as previously defined [22]. Each dot represents one patient, colors code for the distinct groups, as indicated. **B.** *De Finetti* like diagram illustrating the sub-classification of an independent ccRCC dataset (GSE22541) according to the Beleut *et al*. 2012 rules. The color code per patient defines its CTP assignment as identified in A. **C.** Control *de Finetti* like diagram, in which Self Organizing Maps (SOM) have been applied in classifying GSE22541 ccRCC primary tumors for comparison. The color code per patient defines the “CTP” to which the respective tumor would belong according to SOM. **D.** *De Finetti* like diagram illustrating the classification of normal renal tissues of GSE53757 according to identified CTPs. Note the weak correlation of individual samples with profile vectors, leading to mostly a central clustering of analyzed samples. **E.** *De Finetti* like mapping of a breast cancer dataset (GSE12093) and **F.** a Lymphoma dataset (GSE34771) according to RCC-CTPs. Note the increased scattering of individual patients in E and F when compared to control.

We visualized achieved results by means of a *de Finetti* like mapping (23, 25) (Fig 4A) to better highlight the potential distances for each patient from the respective cluster centroids defined as A, B and C. Next, we classified an independent set of clear cell RCCs (ccRCC) (GSE22541), resulting in three similar CTP clear cell RCC cohorts as shown in Fig 4B.

To further solve the question whether other clustering technologies could also have been used as a potential starting point for CTP identification, we utilized Self Organizing Maps (SOM) [26] for clustering of GSE22541 and compared achieved results with the classification shown in Fig 4B (Fig 4C). Result overlap of both technologies was 75%, suggesting that CTPs can be identified independently of the data set GSE19949 which was the starting point of our observations and calculations. The similar outcome with the two clustering technologies used suggests the presence of distinct CTPs in different sets of RCC tumors.

As control, we applied this classification rule on a set of normal renal tissues (GSE53757) but also on other healthy tissues derived from different organs or anatomic body parts as annotated in GSE1133 and GSE2361. In contrast to RCC, healthy controls remained grouped around the center of the *de Finetti* like mapping (Fig 4D and Fig B in S1 Text) confirming RCC specificity of identified CTPs.

To better investigate cancer specificity in general, we also strictly applied identified RCC-CTPs on one dataset of breast cancer (Fig 4E) and one dataset of lymphoma (Fig 4F), respectively. Despite potential cancer-specific background, resulting *de Finetti* like mappings still illustrate an increased scattering of individual patients as compared to control mappings, thus strengthening the assumption of general cancer specificity rather than RCC specificity of proposed CTPs.

Additionally, we investigated the average correlation of all patients assigned for a particular CTP (S1 Text). For every CTP of a data set we collected all contributing patients and their correlation values with the respective CTP and calculated the average. The results may be interpreted as “cluster diameter” estimate. For RCC, the correlation was highest with 0.439; 0.476; 0.413 for CTP-A, CTP-B and CTP-C, respectively. For the other tumor types, these values ranged between 0.14 and 0.18, whereas for control normal tissues these values ranged between 0.08 and 0.14, much lower than for tumors. This difference between normal and tumor tissue was highly significant (p-value = 2*10^−6^). The direct comparison of the normal and tumor tissue samples of the renal data set GSE53757 (0.08 vs. 0.14, respectively) was also significant (p-value = 5*10^−6^). We intentionally omitted the SOM clustered data from GSE22541 from this averaging since the independent SOM clustering introduced additional positional scatter. Detailed results for each GSE dataset and corresponding CTPs are listed (Table E in S1 Text).

### Transferring the CTP model established for RCC to other cancer types

By transferring this RCC based model to gene expression data of other cancer types, we aimed to regroup other cohorts according to conditions homologous to RCC CTP groups A, B and C. The CTP model and its transfer to other cancer types occurred as illustrated in Fig 2H–2J. Resulting CTP subgroups of different cancer types were also correlated with associated clinical data where available. We calculated respective CTPs for all cancer types from published studies as denoted in S1 Table and compared them with reference RCC-CTPs. Highest Pearson correlation with one of the three RCC-CTPs assigned a patient either to CTP group A, B or C.

### CTPs are not RCC-specific and commonly exist in cancer transcriptomes

In order to test whether or not CTPs are exclusively related to RCC, we performed a reverse transfer of the CTP concept (S1 Text). Data from the breast cancer study GSE2603 was used to generate breast cancer-specific CTPs. Samples from this study were clustered with standard unsupervised clustering methods (k-means, SOM) into three clusters, and the respective Kaplan-Meier curves were calculated. The resulting groups were used for transfer to the RCC study GSE19949 with the same procedure utilized for transfer to other cancer types (reverse transfer). The resulting distribution into three groups was 65% identical with the original RCC grouping [22] suggesting a nonrandom finding and a general presence of CTPs detectable in all human cancers.

### CTPs seem to be robust irrespective from gene function and number

**Fig 5:**
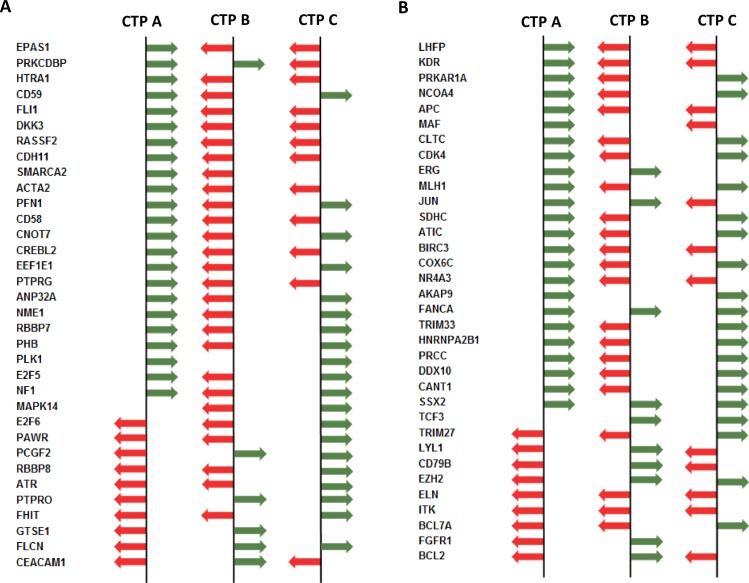
CTP profiles of the different cancer types using randomly picked tumor suppressor or oncogenes. Shown are best descriptors of gene expression deviations from the mean of randomly selected tumor suppressor genes **(A)** and oncogenes **(B)** encompassing different cancer types. For a detailed overview illustrating all expression deviations including genes showing no deviations from the mean, see S4 and S5 Tables. **Red:** relative gene expression deviation is lower than the mean expression, **Green:** relative gene expression deviation is higher than the mean expression. CTPs differ by the overall relative gene expression deviations. The entirety of expression deviations from the mean of genes, not that of one single gene is relevant for affiliating one distinct tumor to CTP-A, -B or -C.

Our efforts for the identification of our CTP concept in human cancer types always included complete sets of genes present on microarray chips. In order to investigate whether indeed all genes or only specific subsets of genes contributed to CTP vector definition, we chose limited numbers of either randomly or functionally defined gene subsets [27] for the generation of CTP target vector profiles using RCC as starting point. We then transduced resulting confined RCC-CTPs to the expression data of the same genes in all other tumor types and compared achieved CTP affiliation of each tumor. We observed for all tumors and tumor types that 700 randomly chosen genes led to an overall similarity of 86 ± 6.6%. This held also true when 716 tumor suppressor genes and 690 oncogenes were chosen for CTP calculation (80 ± 7.7% and 81 ± 8.1% similarity, respectively). Specific results for the individual studies are depicted in S3 Table. Our overall finding suggests that CTPs are non-random and measurable irrespective of gene expression deviations relatively to each other, distinct gene types, gene functions or gene numbers (Fig 5, S4 and S5 Tables). The data shown in S4 and S5 Tables give examples how gene sets with different functions (tumor suppressor genes and oncogenes) are classified into 3 CTPs. The relationship of the CTP groups among different cancer types can be visualized by sorting the values (ascending or descending) obtained from one gene set of one CTP group.

As measuring gene expression is always accompanied by noise, we investigated its impact by performing a simulation. We asked if the separation between CTP-A, -B, and -C as observed in Fig 1 may be entirely or to large part due to noise. In the simulation, we thus assumed that for all patients the deviation from the average expression value of each probe set is just the result of a random fluctuation. The distribution of the deviations is assumed to be normal for every probe set. The subsequent discretization of the values, calculation of the CTP profiles and the ensuing assignment of the virtual patients to a particular CTP was performed according to the protocol, as well as the calculation of the “virtual cancer” specific profile. A random subset of 716 genes was finally picked to mimic the effect of specific gene selection as in the tumor suppressor or oncogene examples.

For the simulation, we generated 14 virtual cancer patient groups with 50 patients each, similar to the experimental situation.

Correlating all CTP-A profiles with each other across different virtual cancer types, and similarly for CTP-B and -C, yielded average correlations of less than 0.02. Calculating the same average correlations for the experimental tumor suppressor genes or oncogenes yielded average correlations between 0.7 and 0.76. The probability that these high correlations between CTPs across different cancer types were generated by random is infinitesimally small (p-value < 10^−100^). Between the two experimental sets, however, the p-values were much higher (0.04–0.98), indicating that the null hypothesis (generated from the same underlying distribution and not by noise) held as expected.

A similar result was obtained when the different CTPs within a cancer type were compared. As expected, experimental correlation values between different CTPs showed values different from 1, i.e. -0.38 (AB), -0.5 –-0.6 (AC), and -0.37 –-0.51 (BC). Again, these correlations were stable across the tumor suppressor and oncogenes platform (p-values ranging between 0.25 and 0.98), while the simulation yielded average correlations between 0.008 and 0.21. This differed significantly from the experimental values (p-value < 0.001).

In summary, these simulations demonstrate that random effects like mere fluctuations of expression values could not explain the high correlations between CTPs across and within different tumor types.

## Discussion

The hallmarks of cancer imply that all cancers follow specific biological concepts which form the basis of malignant behavior. Therefore, we speculated that such biological concepts among cancers relate closely to each other and may be visualized by gene expression patterns. Our hypothesis originated from the detection of three gene expression outputs in renal cell carcinoma (RCC), which appeared to be independent from pathological parameters and correlated with patient outcome [22]. In an attempt to further support this hypothesis we designed an algorithmic model, which enabled us to classify Cancer Transcriptomic Profiles (CTP). The initial experiment on RCC was performed on Affymetrix arrays. In order to transduce the algorithmic rule on other cancer types in the first step, we focused on studies performed using Affymetrix microarrays and screened GEO for surgically treated tumor cohorts with associated progression data and identified 55 studies. Being aware of additional useful meta-analysis on different platforms, we noticed that all these platforms lack a proper key to translate their annotations to each other or to Affymetrix or Illumina or even present entirely different technologies such as RNAseq. By focusing on a single platform, we could exclude all additional factors and potential artifacts due to the chip related technical differences. In one instance, however, we compared the Affymetrix outcome with results obtained from Illumina data to demonstrate that the observed results are independent of the gene expression platform chosen. We selected Illumina for this comparison as this platform has about 6000 probes in common with Affymetrix with equal annotations, thus enabling the partial transfer as performed in our text appendix. The generalizations found across tumor boundaries could thus be safely attributed to the underlying tumor biology.

In this study, we analyzed gene expression data obtained from 8397 samples. Sixteen of 55 datasets accounting for 4177 out of the 8387 tumors were from breast cancer, which currently presents the most frequent tumor type in the GEO repository. We detected three different Cancer Transcriptomic Profiles (CTPs) that are recurring in 13 different cancer types. These CTPs suggest molecular relationships among human cancers.

Absolute gene expression values with subsequent associated gene enrichment technologies have been used in many gene expression studies. In keeping with this, we first used a similar approach and detected 3 groups in RCC by two-way non-supervised hierarchical clustering [22]. Our subsequent goal was to find a mathematic approach, an algorithm, for depicting those 3 groups. According to our opinion, this was best achieved by discretization of gene expression data we describe here. The method we have chosen is *per se* not novel, but to our knowledge, its conceptual application to characterize mathematically the 3 CTPs has not been used and published so far. A closer look at the expression profiles of all genes (Fig A in S1 Text) clearly shows that the expression levels of the genes in each CTP group are equally distributed. CTP groups are not characterized by expression patterns of a specific set of genes (e.g. high *versus* low) which differ, for example, between two organs, tissue/cell types or healthy/diseased tissue. As illustrated in Fig 3, it is the composition of the “expression profiles” yielded from the expression deviations from the mean values from all gene probe sets which defines a specific CTP. According to our opinion one would hardly be able to identify those 3 CTPs by using absolute gene expression levels or a more sophisticated “barcoding” model in which gene expression measurements are banalized to 0 (not expressed) *vs*. 1 (expressed) [28, 29].

Furthermore, a binary approach would only distinguish between expression levels which are either low or high in two groups. With the binary approach one focuses only on low (underexpressed) and high (overexpressed) expressed genes but exclude those genes which are more or less equally expressed. Our model includes also genes with no or small expression level changes. A closer look on the 3 CTPs clearly demonstrates that these genes provide similar contributions to the CTP profiles as the high differentially expressed genes. As a result, CTPs yielded with a 2-bin system would introduce substantial distortions compared to our CTPs.

These three CTP groups first detected in RCC and confirmed by the discretization method, were transferable to 12 additional cancer types. We developed the algorithm applied to address various challenges encountered in transcription analysis, so that individual expression fluctuations per gene or probe set were neutralized. Subsequent to the standard GCRMA normalization step for Affymetrix gene expression chips (S1 Text), we normalized and scaled the expression values of the entire patient set gene-wise. Subsequently we discretized the scaled expression values into 3 levels (S1 Text). By using our -1,0,1 approach of discretization, which aimed at turning the continuous and multi-parametric data into this finite number of discrete elements, we were able to not only facilitate ensuing computations, but also reduce data noise. Clustering the discretized data of the RCC study GSE19949 for 3 Cancer Transcriptome Profiles (CTP-A, -B, -C) with different methods (e.g., k-means, SOM) and an independent classification based on histological parameters [22], yielded almost identical results. Elements of our approach relate to methods applied earlier, such as Linear Discriminant Analysis, Significance Analysis of Microarrays [30] or shrunken centroids [31]. One of the most relevant advantages is that this method is invariant against differences in expression levels resulting from different tissues of origin. It thus may present a novel method enabling the detection of pan-cancer signatures.

According to our mathematical approach, CTPs are a reappearing pattern throughout the cancer transcriptomes. Notably, different clustering technologies, such as k-means or SOM [26, 32], applied after discretization generated similar results. The robust nature of a defined CTP in a tumor was also demonstrated when we investigated the average correlation of all patients assigned for a particular CTP. Despite potential biases which may be caused when calculating with data sets generated from different patient cohorts in different laboratories, the difference of the correlation values obtained from normal and tumor tissue was highly significant. Even the reverse transfer of breast cancer-specific CTPs to RCC resulted in 3 groups which were 65% identical with the original RCC subgrouping further supporting the existence of CTPs in different cancers.

It is of note that due to the very limited availability of samples from normal (control) tissue published in GEO, it is currently not possible to define the particular threshold between normal and tumor tissue. We noticed that the studies mostly consist of disease sample collections with no corresponding healthy counterparts. Therefore, a healthy/disease threshold could not be yielded, unless by including matched pairs of affected and non-affected tissue samples in a sufficiently large amount. The *de Finetti*-like visualizations demonstrate, however, that normal samples located closer to the triangle center than tumor samples. At the center, the correlation with any of the CTP was lowest, pointing to a lower CTP differentiation in normal tissue samples. Being limited to the present situation, however, we can only state, as shown in the results part, that the defined calculated differences are highly significant (p-value = 2*10^−6^) and are not occurring due to randomness.

Finally, CTPs still remained stable with sets of only several hundred genes, whether randomly or non-randomly selected. Therefore, we conclude that i. the expression status of every single gene is important to contribute to a CTP and ii. the expression profile of a minimum set of genes is required to yield a CTP.

As the CTPs of our RCC indicated correlation with patient outcome, we calculated Kaplan Meier survival plots using all survival data sets from different cancer types that were available in the GEO database. The results were, however, difficult to interpret (data not shown). Some survival plots for sarcoma, breast and lung cancer were similar to those obtained from RCC. Other survival plots showed no associations or, as in the case of ovarian cancer, even contrasting patterns. This data strongly suggest that the analysis of survival data sets from different patient cohorts (S1 and S2 Tables) generated from different research groups, as well as the use of different assay and standardization methods may cause dataset bias and inter-dataset noise. The GEO database presents to our knowledge the major resource for researchers for getting access to clinical survival data. Based on our experience its use for analyzing the prognostic value of molecular markers in one specific tumor type or in different cancers is rather limited. However, this tool is ideally suited to perform comprehensive analysis to detect potential associations between molecular signatures and cancer.

## Conclusions

We present a novel model that can be applied to identify cancer-specific gene expression profiles. It has been widely accepted that each tumor has developed its own individual molecular landscape. The entirety of molecular events leading to a malignant tumor affects always the same biological concepts described in the hallmarks of cancer. We believe that the CTPs identified by us represent the molecular outputs which exist in all human cancers. However, more in-depth investigations with larger and better defined cancer patient cohorts are needed to support our CTP concept for cancer biology but also as possible additional tool for cancer prognosis.

## Supporting Information

S1 Table All studies considered for CTP affiliation. (XLSX)

S2 Table Clinico-pathological parameters per CTPs. (XLSX)

S3 Table CTP affiliation and similarity by considering randomly chosen genes. (XLSX)

S4 Table CTPs and Tumor Suppressor Genes in different cancer types. (XLSX)

S5 Table CTPs and Oncogenes in different cancer types. (XLSX)

S1 Text Additional methods, results and figures. (DOCX)
