# Supplementary material for: Discretization of Gene Expression Data Unmasks Molecular Subgroups Recurring in Different Human Cancer Types
Source: PLoS One. 2016 Aug 18;11(8):e0161514. doi: 10.1371/journal.pone.0161514 (PMC4990327; doi:10.1371/journal.pone.0161514)
Supplement: S1 Text — (DOCX) [file pone.0161514.s006.docx]

**Index**

[1 Data 2](#_Toc457846495)

[1.1 Renal cell carcinoma data set 2](#_Toc457846496)

[1.2 Data of other cancer indications 3](#_Toc457846497)

[2 Data preprocessing 5](#_Toc457846498)

[3 Algorithm – generation of CTPs 6](#_Toc457846499)

[3.1 Determination of CTP for unknown data set 7](#_Toc457846500)

[4 Testing for stability and reliability 8](#_Toc457846501)

[4.1 RCC cross validation 8](#_Toc457846502)

[5 Transfer 9](#_Toc457846503)

[5.1 Transfer to breast cancer studies 9](#_Toc457846504)

[5.2 Transfer to HG-U133B 10](#_Toc457846505)

[5.3 Transfer to other cancer indications 10](#_Toc457846506)

[6 Reverse Transfer of CTPs 10](#_Toc457846507)

[7 Different microarray platforms and chip types 11](#_Toc457846508)

[8 Visualization of Cancer Transcriptomic Profiles 12](#_Toc457846509)

[9 *De Finetti* like mappings of other normal tissues 13](#_Toc457846510)

[10 Quantification of *de Finetti* like mappings 14](#_Toc457846511)

# Data

## Renal cell carcinoma data set

The initial hypothesis for classifying patients according to Cancer Transcriptomic Profiles (CTP) was first suggested on a renal cell carcinoma (RCC) gene expression data set (GSE19949). The results based on genome wide gene expression data, Single Nuclear Polymorphism (SNP) technology and immunohistochemistry using a tissue microarray (TMA), were published: Beleut M, Zimmermann P, Baudis M, Bruni N *et al. “Integrative genome-wide expression profiling identifies three distinct molecular subgroups of renal cell carcinoma with different patient outcom*e.” **BMC Cancer** 2012 Jul 23;12:310. <http://www.biomedcentral.com/1471-2407/12/310>

Gene expression data from 98 renal cell cancer samples had originally been measured employing the Affymetrix^®^ HT_HG-U133A microarray (Platform GPL3921, <http://www.ncbi.nlm.nih.gov/geo/query/acc.cgi?acc=GPL3921>). The samples had been categorized in groups A, B, or C according to an independent procedure described in Beleut *et al*. The gene expression data is stored at the NCBI GEO (Gene Expression Omnibus) online repository under the accession number GSE19949. The gene expression data had been measured on two sample carriers with BI cohort, comprising 33 samples, and BII cohort, comprising 65 samples.

Survival data was available for 89 patients of the initial 98 RCC patient cohort.

## Data of other cancer indications

All gene expression microarray data used in this publication is publically available and can be retrieved from the GEO repository. All available, associated clinical annotations containing survival data and molecular- or pathological criteria were used. Table A in S1 Text presents a list of the data sets where pathologic parameters were available. Studies with the associated endpoint “overall survival” were used for Kaplan-Meier curve calculation but not shown, as discussed, in the manuscript.

The data sets comprise Affymetrix^®^ HG-U133A (GPL96) and / or HG-U133B (GPL97) and / or HG-U133Plus2 (GPL570) arrays. In one instance, Illumina^®^ HumanHT-12 V3.0 expression beadchips (GPL6947) were used as control.

**Table A:** A systematic overview of all studies with progression data grouped into CTPs.

All studies are published on the GEO database and comprise associated clinical data for most patients. Outlined are the GSE numbers, the cancer indication, endpoints as published within the studies as well as the total number of patients with their assignment to CTP-A, CTP-B or CTP-C, respectively. GSE 24450 was run on Illumina^®^ arrays and was used as control, all other studies were performed on Affymetrix^®^ human gene chip arrays.

# Data preprocessing

Affymetrix® HT_HG-U133A, HG-U133A, HG-U133B, and HG-U133Plus2 CEL files were preprocessed using packages from BioConductor [(http://www.bioconductor.org/).](http://www.bioconductor.org/) They were normalized using the “affy” package version 1.42.2 comprising rma(affybatch) for RMA normalization, and expresso(affybatch, bg.correct=FALSE, normalized.method=”invariantset”, summary.method=”liwong”, pmcorrect.method= ”pmonly”) for dCHIP normalization, as well as “gcrma” package version 2.36.0 comprising gcrma(affybatch) for GCRMA normalization. Annotations were loaded from the annotation package “annotate” version 1.42.0 and “AnnotationDbi” version 1.26.0.

In the analysis of HG_U-133A arrays the homologue probesets from HG_U133Plus2 arrays were added. The same procedure was applied to the analysis of HG_U-133B arrays.

For the Illumina® array HumanHT-12 V3.0 expression bead chip, the log expression values were used for comparison with Affymetrix^®^ arrays.

The reference data set GSE19949 contained 65 (BII cohort) gene expression samples using Affymetrix^®^ HT_HG-U133A microarrays, 42 of which had been classified as A, 9 as B, and 14 as C. The BI cohort contained 33 samples, 7 of which had been classified as A, 14 as B and 12 as C. Normalizations were performed separately for GSE19949 BI (GSM498450-GSM498469 and GSM498529-GSM498541) and BII (GSM498470-GSM498528 and GSM498542-GSM498548) or, for comparison, jointly for BI and BII.

The log values of the Illumina^®^ (only GSE24450) data were used for calculation. To account for experimental variance, normalizations with all three methods mentioned were performed separately for BI and BII subsets of the RCC GSE19949 data set, or, for comparison, jointly for BI and BII.

# Algorithm – generation of CTPs

The reference data set consists of samples from GSE19949 for RCC, for which classification into groups A, B, and C was carried out. The reference data set BII contained 65 gene expression samples using Affymetrix^®^ HT_HG-U133A microarrays, 42 of which had been classified as A, 9 as B, and 14 as C. Corresponding figures for BI are 33 data sets, 7 of which had been classified as A, 14 as B, and 12 as C.

The method comprises the following steps:

Profiling from reference data set

Let xij be the expression values for genes i = 1, 2, …, m and samples j = 1, 2, … n (n = 65) for the BII reference set, and let


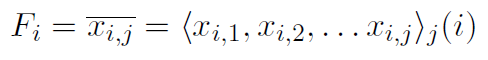


be the mean of the distribution of expression values per gene for all patients. We assign values -1 (below average expression), 0 (close to average expression), and 1 (above average expression) for expression values in the 1^st^, 2^nd^ and 3^rd^ tertile, respectively, so that

according to the position of the corresponding xij in the respective tertile.

The rationale for discretizing the deviation from average is that absolute deviations are subject to experimental artefacts, which differ from sample to sample. For low expression values, background noise may alter the deviation value significantly. After classifying deviations as above, close to, and below average (or 1, 0, and -1), these artifacts are reduced. Next, we calculate the mean values yai, ybi, yci separately for all samples adhering to a particular group:

Hence we have obtained a profiling vector (CTP) for every group A, B, C.

## Determination of CTP for unknown data set

For the new data set, individual vectors

are calculated in the same way as in 1). Next, the correlation between Yj and Ya, Yb, and Yc is calculated. The patient j is then assigned to the CTP with the highest Pearson correlation value, thus

# Testing for stability and reliability

## RCC cross validation

GSE19949 BI was taken as training set and BII as validation set, and *vice versa*. It was calculated how well samples from the one set could be predicted correctly from the other. Moreover, to reflect the influence of artefacts, the validation was performed using different normalizations.

**Table B: Training and prediction quality for different models**.

The models were named m1 if BI dataset was used for training and m2 if BII was used for training. The normalization method (GCRMA, RMA, dCHIP) was part of the model. The numbers represent percent of samples correctly assigned to the original CTP A, B, or C. The original assignment for RCC was used as reference. For example, the highlighted cells refer to the model m2 where BII normalized with GCRMA was taken as training set to predict BI normalized with all three normalization methods (Table B in S1 Text). Best results are obtained with the highlighted configuration, where model m2 (based on BII as the training set, normalized using GCRMA) obtains good classification results for BI (used as the validation set) despite strong experimental artefacts.

# Transfer

## Transfer to breast cancer studies

The RCC-CTP-based models were tested on breast cancer data. The consistency of the results was checked by employing different normalizations for the breast cancer data.

**Table C:** **Stability of prediction using different models**.

Models m1 and m2 with their respective normalization methods were tested against each other on breast cancer data from GSE 11121. The numbers represent the percentage of identical assignment to A, B, C for the respective model and normalization pair. For example, for the highlighted cell the transfers performed with m1 and RMA normalization, and m2 and GCRMA normalization, still overlap each other at 65%.

Example for the robustness of the transfer process: The breast cancer data set GSE 11121 was normalized with dCHIP, the RCC data with all 3 normalization methods resulting in very similar predictions (Table C in S1 Text).

## Transfer to HG-U133B

Using the CTP assignment generated in 5.1 for breast cancer study GSE1456 samples based on the HG-U133A microarrays, the HG-U133B arrays available for the same patients were used to calculate CTP profiles for HG-U133B. These profiles were taken to classify samples for other studies containing HG-U133B microarrays. It was found that the survival estimate split between the CTPs was maintained.

## Transfer to other cancer indications

Publically available data from NCBI GEO of the different cancer indications were downloaded (S1 Table) and calculated. Only studies with associated clinical data (overall survival) were considered for Kaplan-Meier analysis (Table A in S1 Text).

In some studies, multiple microarrays per patient are measured.

Studies were used for assignment of CTP according to the method described in chapter 3.

# Reverse Transfer of CTPs

To further test the robustness of the algorithm, a reverse calculation was performed. To this end, the data from one breast cancer study (GSE2603) were preprocessed (normalized with GCRMA, scaled and transformed to the (-1,0,1) scale) and random assignments of CTP A, B, C were made 10 times. From those random assignments, “Kaplan-Maier curves” were calculated and the random assignment with the most significant p-value, e.g. split between survival estimates, was taken for further analysis.

From this particular choice, CTP profiles for the BC were calculated and used to classify the B2 GSE19949 data. It was found that CTPs for RCC generated by this backward calculation were to 74% identical with the original CTP assignment.

Similar results (65% similarity) were obtained when the BC data were grouped in three CTPs by means of unsupervised clustering (k-means and SOM) instead of random CPT assignments.

# Different microarray platforms and chip types

When different microarray platforms are used (e.g., Illumina^®^ and Affymetrix^®^), the number of probe sets / genes used is restricted to those which can be unambiguously mapped, i.e., where a single probe set of one chip type can be mapped to a single probe set of the other. In the case of GSE19949 GPL96 to GSE24450 GPL6947, 5111 probe sets were found to map unambiguously. The common probe sets for the Affymetrix^®^ arrays used in the calculations are shown in Table D in S1 Text.

|  | HT_HG-U133A | HG-U133A | HG-U133B | HG-U133Plus2 |
| --- | --- | --- | --- | --- |
| HT_HG-U133A | 22277 | 22277 | 162 | 22277 |
| HG-U133A |  | 22283 | 168 | 22277 |
| HG-U133B |  |  | 22645 | 22369 |
| HG-U133Plus2 |  |  |  | 54675 |

**Table D: Number of common probes for different Affymetrix^®^ microarrays used in the calculations.**

# Visualization of Cancer Transcriptomic Profiles

Fig A in S1 Text shows the variation of the CTP profiles for the RCC BII data set. To this end, the probe sets were sorted according to their respective CTP A profile value, which means they smoothly increase. The insets show zooms of high, low, and medium CTP A profile values (red). CTP B is shown in blue, CTP C in green. As expected, the values for B and C fluctuate much after sorting for A, and show a slight anticorrelation trend to each other and to A. Sorted for B or C, the graphs look similar.


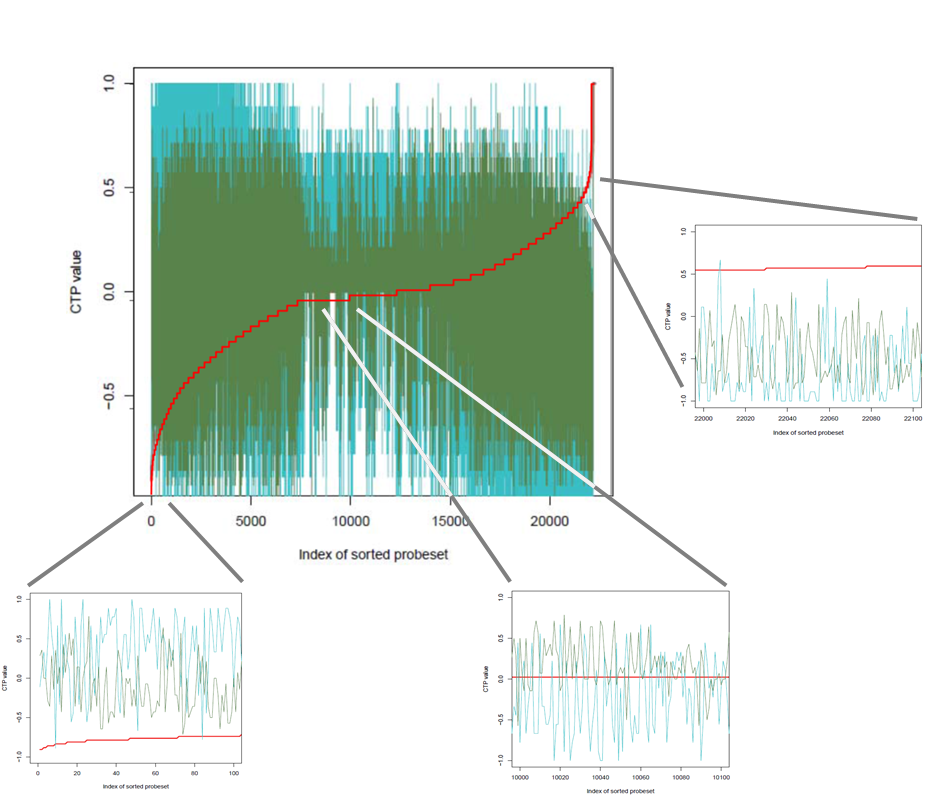


**Fig A: Shown are the CTP values in increasing order for A, as well as B and C in the same order**. Thus A (red) is smoothly ascending with the Index, while B (blue) and C (green) are fluctuating. The inserts show zooms for low, medium, and high CTP values for A. Respective index ranges are 1-100, 10,000 – 10,100, and 22,000 – 22,100.

# ***De Finetti* like mappings of other normal tissues**

In order to investigate tumor specificity, the CTP classification rule was applied on other human and normal tissues derived from different organs then renal, as annotated in GSE1133 and GSE2361.


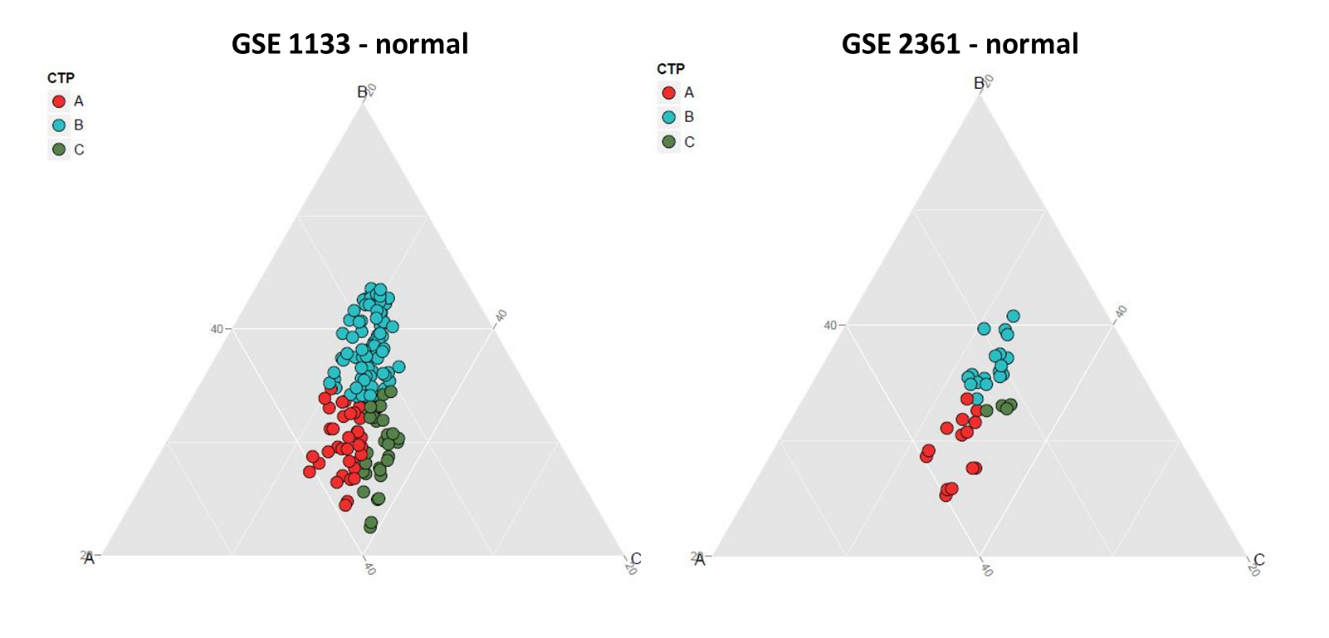


**Fig B: *De Finetti* like mappings of normal tissues according to identified RCC-CTPs**.

Samples remain largely grouped around the centers when compared with tumors, pointing towards a cancer specific nature of identified CTPs (Fig B in S1 Text).

# Quantification of *de Finetti* like mappings

**Table E: Quantification of individual CTPs in shown *de Finetii* like mappings, tumors *vs.* controls.**

As expected, the correlation of GSE19949 with CTPA, CTPB and CTPC is highest. The tumor datasets, Breast Cancer (GSE12093), Lymphoma (GSE34771) and one additional RCC dataset (GSE 22541) correlate better with defined CTPs as compared to the used controls of “normal” tissue (GSE2361, GSE1133, GSE53757), respectively (Table E in S1 Text).
